# A framework for developing, validating, and utilizing automated measures of order errors using the retract-and-reorder methodology

**DOI:** 10.1093/jamiaopen/ooag165

**Published:** 2026-09-09

**Authors:** Pooja Reddy Spector, Anne Grauer, Jerard Z Kneifati-Hayek, Benjamin L Ranard, Jo R Applebaum, Yelstin Fernandes, Hojjat Salmasian, William N Southern, Clyde B Schechter, Gregory Hruby, Joseph T Cooke, Deepa Kumaraiah, Baruch Fertel, Jason S Adelman

**Affiliations:** Department of Pediatrics, Columbia University Irving Medical Center, New York, NY 10032, United States; Department of Medicine, Columbia University Irving Medical Center, New York, NY 10032, United States; Department of Medicine, Columbia University Irving Medical Center, New York, NY 10032, United States; Department of Medicine, Columbia University Irving Medical Center, New York, NY 10032, United States; Department of Medicine, Columbia University Irving Medical Center, New York, NY 10032, United States; Department of Medicine, Columbia University Irving Medical Center, New York, NY 10032, United States; Department of Medicine, Brigham and Women’s Hospital/Harvard Medical School, Boston, MA 02115, United States; Department of Medicine, Albert Einstein College of Medicine, Montefiore Medical Center, Bronx, NY 10467, United States; Department of Family and Social Medicine, Albert Einstein College of Medicine, Bronx, NY 10467, United States; Department of Quality and Patient Safety, NewYork-Presbyterian Hospital, New York, NY 10032, United States; Department of Medicine, NewYork-Presbyterian/Queens Hospital, Queens, NY 11355, United States; Department of Medicine, Columbia University Irving Medical Center, New York, NY 10032, United States; Department of Quality and Patient Safety, NewYork-Presbyterian Hospital, New York, NY 10032, United States; Department of Quality and Patient Safety, NewYork-Presbyterian Hospital, New York, NY 10032, United States; Department of Emergency Medicine, Columbia University Irving Medical Center, New York, NY 10032, United States; Department of Medicine, Columbia University Irving Medical Center, New York, NY 10032, United States; Department of Quality and Patient Safety, NewYork-Presbyterian Hospital, New York, NY 10032, United States

**Keywords:** computerized provider order entry systems, health information technology, medical errors, medication errors, patient safety, retract-and-reorder (RAR)

## Abstract

**Objective:**

Despite the recognized need to leverage health information technology (IT) to facilitate error detection, there are few health IT safety measures in use and limited resources available to guide the conceptualization, development, and testing of new measures. Audit log data provide a rich source of clinician behaviors within the electronic health record (EHR), which can be used to develop health IT safety measures.

**Materials and Methods:**

With illustrative examples, we describe a framework for the development and evaluation of novel order error measures by employing the Retract-and-Reorder (RAR) methodology, which uses audit log data to identify actions indicative of potential order errors.

**Results:**

Topics covered include the following: (1) insight into the value of large data repositories generated during clinical care for creating automated health IT safety measures, (2) the principles and theoretical model that underly the ability of RAR measures to detect order errors, (3) an experience-based framework for the conceptualization, implementation, validation, and optimization of new RAR measures to detect order errors, and (4) applications of RAR measures to guide understanding of health IT safety events.

**Discussion:**

This report is intended for informatics and health services researchers and healthcare system leadership who seek to utilize EHR audit log data to identify and quantify order errors in EHR systems.

**Conclusion:**

The methodology described can be applied to develop automated measures to detect a range of order error types, examine the epidemiology, and investigate the root causes of order errors in near-real-time, as well as rigorously evaluate the impact of preventive interventions.

## Introduction

The Institute of Medicine report, *Health IT and Patient Safety: Building Safer Systems for Better Care*, raised awareness of patient safety risks introduced by health information technology (IT).^1^ The report noted a lack of systems for quantifying health IT safety risks and called for the Agency for Healthcare Research and Quality (AHRQ) and the National Library of Medicine to fund the development of measures to reliably assess the state of health IT safety and monitor improvement. Additionally, the National Quality Forum (NQF) report, *Identification and Prioritization of HIT Patient Safety Measures*, encouraged the use of electronic health record (EHR) data to facilitate error detection and measurement to improve the safety of health IT systems.^2^ Despite the recognized need for validated health IT safety measures in research and practice, there are few such measures in use and limited resources available to guide the conceptualization, development, and testing of new measures.^3–6^

The near-universal transition to EHRs has created an opportunity to develop health IT safety measures by leveraging the large volume of electronic data generated in clinical care. The EHR audit logs, which contain records of user activity, have demonstrated utility for error detection.^7^^,^^8^ Details of electronic orders extracted from audit log data were used to develop the Wrong-Patient Retract-and-Reorder (RAR) measure, the first health IT safety measure endorsed by NQF (Measure no. 2723) in 2015 and more recently in the Safety Assurance Factors for EHR Resilience (SAFER) guidelines as a core tool for monitoring and reducing wrong-patient order errors.^9^^,^^10^ The Wrong-Patient RAR measure identifies order errors that follow a specific sequence of events: one or more orders placed for a patient, retracted (canceled) by the same clinician within 10 minutes, then reordered by the same clinician for a different patient within the next 10 minutes.^3^^,^^5^ Using near-real-time phone interviews with clinicians to verify the errors, the automated Wrong-Patient RAR measure proved to be a valid and reliable method for detecting wrong-patient electronic orders.^5^ Use of this measure is recommended by the Office of the National Coordinator for Health Information Technology (ONC) and Joint Commission to actively monitor EHR safety.^10^^,^^11^ The Wrong-Patient RAR measure has been used to examine the epidemiology of wrong-patient orders across clinical settings and to demonstrate the effectiveness of interventions for preventing these errors,^5^^,^^12–20^ one of which led to the Joint Commission National Patient Safety Goal to prevent misidentification errors among newborns.^12^^,^^21^

The RAR measures can be used to describe the epidemiology of order errors, investigate their root causes, and provide the outcome metric to test patient safety interventions. These measures overcome many of the limitations of traditional methods used to quantify medical errors, such as chart review and voluntary reporting.^22^^,^^23^ Retract-and-reorder measures have several advantages: (1) objectivity, eliminating biases introduced by voluntary reporting and the lack of documentation of patient safety events in the medical record; (2) immediacy, capturing and providing the unique opportunity to investigate patient safety events in near-real-time; (3) automation, systematically identifying patient safety events and providing a robust volume of outcomes to power intervention studies; and (4) generalizability, applicable across EHRs, healthcare systems, and settings.

**Figure 1.**
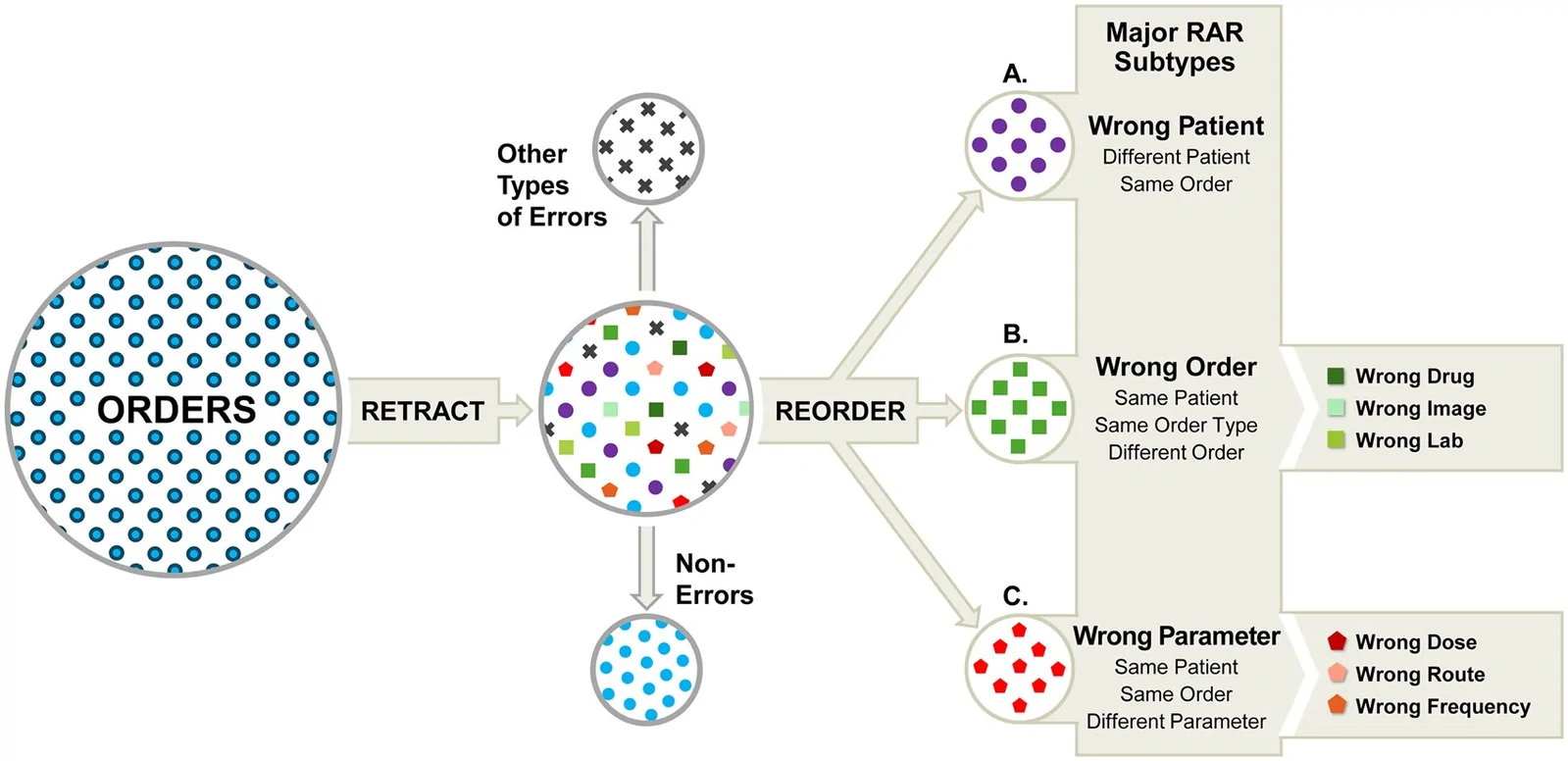
Retract-and-reorder (RAR) theoretical model. The RAR model posits that an order placed, rapidly retracted, and reordered with an element of the order changed is likely an order error. The element of the order that changed indicates the error type. Analysis of orders using the RAR model identified 3 major subtypes of RAR events. (A) Wrong patient, in which an order is placed for a patient, retracted by the same ordering clinician, then reordered for a different patient by the same clinician. (B) Wrong order, in which an order is placed for a patient and retracted by the same ordering clinician, who then reorders a different order of the same type for the same patient (eg, different drug, different imaging test or different lab test). (C) Wrong parameter, in which an order is placed for a patient, retracted by the same ordering clinician, then reordered for the same patient by the same clinician with a single parameter of the order changed (eg, different dose, different frequency).

The Wrong-Patient RAR measure demonstrated proof of principle for the RAR theoretical model,^5^^,^^12–14^^,^^18^ which may be applied to capture many other types of order errors. The RAR model posits that a rapidly retracted order represents an error, and the order placed immediately after the retraction, with an element of the initial order changed, is the corrected order. The element of the order that changed indicates the error type (Figure 1). The RAR theoretical model is supported by prior studies demonstrating that rapidly retracted orders are likely order errors.^3^^,^^5^^,^^24^^,^^25^

Analysis of electronic orders from EHR audit log data at our large healthcare system further supported the potential of additional error measures using the RAR theoretical model. Over a 1-year period, clinicians placed a total of 21 401 986 orders, of which 385 965 orders (1.8%), or more than 1000 per day, were retracted within 30 minutes (Table 1).^26^ Notably, wrong-patient RAR events represented a small proportion (3.6%) of all retracted events. Among all retracted orders, 112 114 (29.1%) were other types of RAR events, that is, rapidly reordered by the same ordering clinician with one or more elements of the order changed. This demonstrated the potential to create new RAR measures to capture a range of different error types.

**Table 1.** Distribution of retracted orders within 30 minutes in a 1-year period (2018) highlighting the potential of new RAR measures to capture diverse error types.

Order type	Total orders		Retracted orders		RAR orders^a^	
	*N*	%	*n*	%	*n*	%
Overall	21 401 986		385 965			
Wrong parameter					112 114	29.0
Wrong patient					11 537	3.0
Wrong order/error without reorder/non-errors					262 314	68.0
Non-medications	15 939 957	74.5	126 794	32.9		
Wrong parameter					26 577	21.0
Wrong patient					5773	4.6
Wrong order/error without reorder/non-errors					94 444	74.5
Medications	5 462 029	25.5	259 171	67.1		
Wrong parameter					81 506	31.4
Wrong dose					20 153	
Wrong frequency					12 353	
Wrong route					5556	
Wrong PRN					1626	
Wrong duration					418	
Other/unknown wrong parameter					41 400	
Wrong patient					5764	2.2
Wrong order/error without reorder/non-errors					171 901	66.3

a RAR order percentages are proportions of each retracted order subgroup.

Abbreviations: PRN, as needed (pro re nata); RAR, retract-and-reorder.

**Figure 2.**
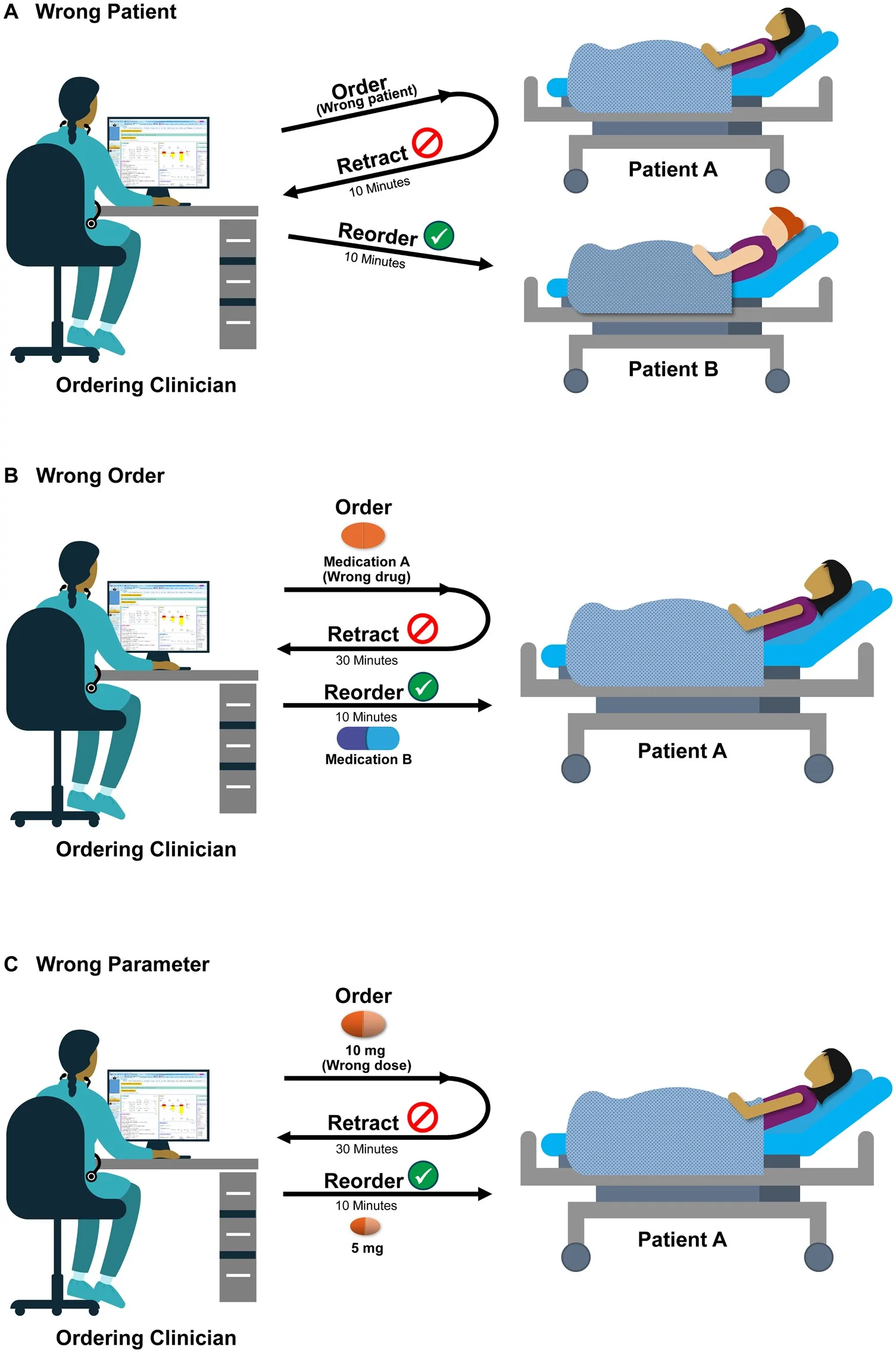
Retract-and-reorder (RAR) sequences. Schematics of the order sequence for the 3 major RAR subtypes. (A) Wrong patient, an order is placed for a patient, retracted by the same ordering clinician, then reordered for a different patient by the same clinician. (B) Wrong order, an order is placed for a patient and retracted by the same ordering clinician, who then reorders a different order of the same order type for the same patient (eg, wrong drug). (C) Wrong parameter, an order is placed for a patient, retracted by the same ordering clinician, then reordered for the same patient by the same clinician with a parameter of the order changed (eg, wrong dose).

The above analysis identified 3 major subtypes of RAR events (Figure 1): (A) Wrong patient, an order is placed for a patient, retracted, then reordered for a different patient by the same clinician; (B) Wrong order, an order is placed and retracted by the same clinician, who then reorders a different order of the same type (eg, different drug, different imaging test, different laboratory test) for the same patient; and (C) Wrong parameter, an order is placed and retracted by the same clinician, then reordered for the same patient with a single parameter of the order changed.  Examples of the sequences of ordering, retracting, and reordering medications for each of these 3 major subtypes of RAR events are displayed in Figure 2.

In this report, based on our experience and prior research, we use illustrative examples to describe a framework for conceptualizing new RAR measures, specifying and implementing queries of audit log data to create the measures, conducting near-real-time phone interviews with clinicians to validate the measures, and applying methods to optimize the performance of the measures. In addition, we review methods to qualitatively and quantitatively analyze the events captured by RAR measures. Our objective is to provide a roadmap to enable others to develop, validate, and utilize novel health IT safety measures, using tools readily available in healthcare settings.

## Framework for developing and validating RAR measures

Our methodology for the development, validation, and utilization of RAR measures includes 5 key components as shown in Table 2: (1) measure conceptualization, (2) measure specification and implementation, (3) measure validation and error investigation, (4) measure optimization, and (5) measure utilization.

**Table 2.** Methodological framework for developing, validating, and utilizing retract-and-reorder measures.

Process	Method
Measure conceptualization	Determine the error type captured by the measure – Literature review – Voluntary incident reporting (internal/external) – Preliminary analysis of EHR data (internal) Determine maximum retract and reorder time intervals
Measure specification and implementation	Design query elements and logic (eg, SQL) Execute near-real-time query (eg, C#, Python) Use API to populate order details into survey platform Generate email notification
Measure validation and error investigation	Conduct interviews with clinicians who triggered RAR events – Emphasize safe space to discuss errors, use non-leading questions Classify events as true positive (measure specified errors) or false positive For true positives, explore the mechanisms that led to error For false positives, explore why the measure triggered the event Calculate positive predictive value
Measure optimization	Analysis of false positives – Revise query to eliminate false positives Use simulated trial and sequential precision and power analyses to determine optimal parameters – Time intervals (time to retract, time to reorder) – Magnitude of change
Measure utilization	Explore health IT safety events – Adapt and execute retrospective query – Qualitative content analysis of reasons for errors – Quantitative analysis of epidemiology of RAR events by encounter, clinician, patient, and order characteristics Evaluate effectiveness of interventions to prevent order errors – Provide sufficient outcome events to power research studies to rigorously evaluate interventions aimed at preventing order errors

Abbreviations: API, application programming interface; EHR, electronic health record; IT, information technology; RAR, retract-and-reorder; SQL, structured query language.

### Measure conceptualization

Based on the RAR model, there are 2 broad decisions to be made when conceptualizing RAR measures: (1) *type of error* the measure aims to capture (ie, what element of the order changed) and (2) *time intervals* from initial order to retraction (retract time) and from retraction to reorder (reorder time). Time intervals impact the number of events captured by the measure and its performance.

#### Determining the error type captured by the measure

Any element of an order can form the basis of a potential RAR measure. To select error types for measure development, we first perform a review of patient safety literature and national databases to identify order error types that are frequently reported, clinically significant, and in need of preventive strategies.^27–31^ Second, we review internal voluntary incident reporting events to identify clinically significant order errors. Finally, we conduct preliminary analyses to determine if a potential RAR measure will generate a sufficient number of events to create a meaningful measure. For example, we focused on developing and validating measures to capture medication order errors (eg, wrong dose, wrong route, wrong frequency), as these errors have occurred in our health system and are a common category of patient safety events and preventable harm reported in the literature.^32–34^ In a demonstrative analysis to evaluate if there would be a sufficient number of RAR events to create meaningful medication order error measures, we determined that of >21 million electronic orders in 1 year in our health system, 67% of the 259 171 orders that were retracted within 30 minutes were for medications (Table 1).^26^ Among retracted medication orders, 81 506 were reordered within 30 minutes and included changes in dose (24.7%), frequency (15.2%), and route (6.8%). In this example, these data supported the viability of medication safety RAR measures and informed our decision to proceed with measure specification, implementation, and validation.

#### Determining maximum retract and reorder time intervals

Prior studies of electronic order error detection methods reported positive predictive value (PPV), defined as the proportion of events determined to be true positives, at varying time intervals.^3–5^ Koppel et al. found that 66% of medication orders canceled within 45 minutes were errors.^3^ When creating new RAR measures, we specify retract and reorder times based on an analysis of internal data, with the goal of maximizing both the volume of events and proportion of retracted orders likely to be errors. For example, in an analysis of retract time up to 12 hours for new RAR measures, more than 50% of order cancellations occurred within 30 minutes, which aligns with prior findings reporting a considerable decrease in PPV for orders canceled after 30 minutes.^3^ When looking at all orders retracted within 30 minutes of being ordered, the mean time from retraction to reorder was 1.4 minutes, which is also consistent with prior studies.^4^^,^^5^

When developing new measures to capture medication order errors, we initially define measures as a sequence in which an order was placed, retracted within 30 minutes, and reordered within 10 minutes by the same ordering clinician with a single parameter of the order changed from the initial order to the reorder. Time intervals are then adjusted to optimize measure performance based on validation results, considering the effect on PPV and total number of events (see *Measure optimization*).

### Measure specification and implementation

#### Identifying RAR events with RAR queries

The RAR measures capture near-miss errors, caught and corrected before reaching the patient. The RAR queries run from a data warehouse or replica server to capture events over a specified time period depending on the purpose. Using a standard database programming language, such as structured query language (SQL), RAR queries extract details of each order (including order type, order name, order date/time, retraction date/time), clinical setting, encounter type, clinician, and patient (Table S1). The query then flags RAR events that meet the definition for the specified error type. Batch orders (ie, flu vaccine for high-risk groups) and system-generated orders (ie, created by computers rather than by humans) are excluded by the query.

When used to validate new measures, RAR queries are run frequently (eg, every 30 minutes) to identify new RAR events that recently occurred. When used to investigate the epidemiology of order errors or evaluate the effectiveness of interventions aimed at preventing order errors, RAR queries are run over months or years (described in detail below).

#### Near-real-time query for measure validation

**Figure 3.**
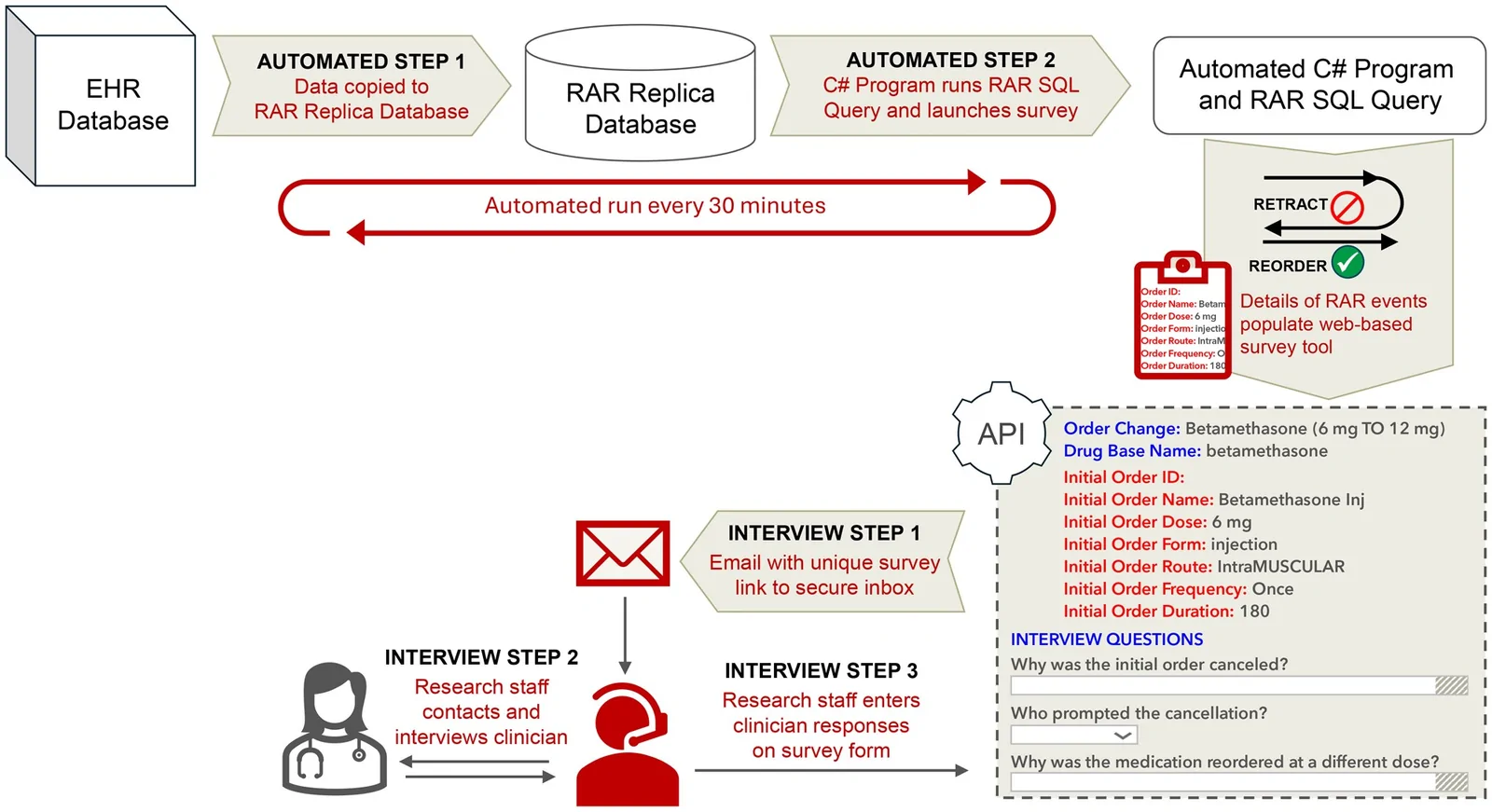
Retract-and-reorder (RAR) data flow for near-real-time measure validation. A batch program automatically runs every 30 minutes and copies all orders and retractions from the EHR database for the prior 30 minutes to an RAR replica database. Next, a C# program runs a structured query language query searching the RAR replica database for any new RAR events in which the reorder that completed the RAR sequence occurred in the prior 30 minutes. Any orders placed, canceled, and then reordered according to the predefined specifications are flagged as RAR events. For RAR events identified by this process, the C# program generates a unique electronic survey in a HIPAA-compliant survey platform to investigate the RAR event. Event details are automatically populated into the survey, including clinician information (name, department, role, contact information), patient information (name, medical record number, age, sex, location), order information for the initial order and reorder (order ID, order name, and order details), and retract and reorder times. The C# program uses the application programming interface to automatically generate and send a secure email with an RAR event-specific survey link to a secure email inbox accessible only by research personnel.

For validation of new RAR measures, we develop and use a system to identify an RAR event in near-real-time, which enables contacting the ordering clinician to assess whether the event was an error (true positive) or false positive, and to investigate why the error occurred or why the RAR query triggered a false-positive event. The data flow for the RAR queries is shown in Figure 3. A batch program automatically runs every 30 minutes and copies all orders and retractions from the EHR database for the prior 30 minutes to an RAR replica database. Next, a C# program runs an SQL query searching the RAR replica database for any new RAR events in which the reorder that completed the RAR sequence occurred in the prior 30 minutes. Any orders placed, canceled, and then reordered according to the predefined specifications are flagged as RAR events. For RAR events identified by this process, the C# program generates a unique electronic survey in a HIPAA-compliant (Health Insurance Portability and Accountability Act of 1996) survey platform to investigate the RAR event. Event details are automatically populated into the survey, including clinician information (name, department, role, contact information), patient information (name, medical record number, age, sex, location), order information for the initial order and reorder (order ID, order name, and order details), and retract and reorder times. The C# program uses the application programming interface to automatically generate and send a secure email with an RAR event-specific survey link to a secure email inbox accessible only by research personnel.

### Measure validation and error investigation

Using near-real-time RAR queries and event-specific surveys, we conduct phone interviews with clinicians to investigate RAR events. Clinician interviews provide a unique opportunity: (1) to verify if events are true positives (measure specified errors) or false positives shortly after they occur, minimizing recall bias and self-reporting bias (measure validation), (2) to investigate underlying reasons for errors for true positives (error investigation), and (3) to investigate why false positives occur to eliminate them from the measure when possible (measure optimization).^3^^,^^5^ Using the measure-specific email and survey link to access event details, research personnel contact the clinician as soon as possible but no longer than 6 hours after the RAR event occurred. After obtaining verbal consent, a brief interview is conducted using a semi-structured interview guide embedded in the electronic survey form to elicit details about the event (Figure S1). Clinicians’ responses are transcribed by the interviewer immediately after the interview. Research personnel undergo training including observation and feedback prior to conducting interviews. To limit disturbances to frontline staff, clinicians who completed or declined an RAR interview within the prior 3 months are excluded from contact.

#### Interview structure and content

To create a safe space to discuss potential errors, we based our interview guide on a framework developed by Schiff et al. to identify reasons for order errors.^24^^,^^35^ This method uses nonleading, open-ended questions to elicit what happened, why the event occurred, and what could have prevented the event (Figure S1). This approach mitigates sensitive requests to admit an error while eliciting reasons why events occur for each error type.

#### Classifying true-positive and false-positive events

For each RAR measure, 2 clinicians independently review interview responses and order details and classify each event as a true positive or false positive. To standardize event classification in our validation studies, we developed a tool with detailed criteria based on a review of patient safety literature,^27–31^^,^^36–40^ expert opinion, and review of pilot validation data. True positives (measure-specified errors) are defined as order changes that capture the intended sequence of events specified by the measure (eg, wrong dose, wrong route, wrong frequency) and were made because placement of the initial order was unintentional, unplanned, due to lack of knowledge, due to failure to follow protocols, or was clinically inappropriate.^37^^,^^40^ In many cases, the error is acknowledged by clinicians or is evident from the description provided. False positives are either non-errors or errors of a different type than the measure intended to capture. From our experience, false positives that are non-errors often result from order changes that are clinically equivalent (eg, due to medication availability, ease of administration, or based on the clinician’s judgment in which both the original order and the changed order are appropriate options), or are order changes that are made because new clinical information becomes available after the initial order was placed (eg, laboratory results, change in clinical status). False positives that are errors of a different type are events in which the query captured errors but not of the type the measure is designed to capture (eg, the wrong-patient RAR measure captured duplicate orders that were canceled, and by happenstance followed by the same order being placed for another patient). Reviewers are instructed to code events in which they are uncertain as false positives for a more conservative estimate of PPV. Classification disagreements are adjudicated by a third clinician. For each measure, inter-rater reliability is assessed using the Cohen’s Kappa coefficient.

#### Positive predictive value

The primary outcome of validation for each measure is PPV, calculated as the number of true-positive events divided by the total number of RAR events investigated during the validation process (true-positive events + false-positive events), along with the exact binomial confidence interval (the Clopper–Pearson interval).^41^

### Measure optimization

After reviewing validation data for each measure, we optimize the performance of each measure by (1) identifying ordering patterns that led to false-positive events and modifying the query to exclude these events and (2) varying measure parameters to maximize the volume of events captured, while maintaining an acceptable PPV.

#### Identifying patterns of false-positive events

Throughout the validation process, we analyze order details and interview narratives to ensure that the captured RAR events meet measure definitions. We also attempt to identify patterns of false positive events that can be excluded by refining query specifications, thereby improving the PPV. For example, for the Wrong-Dose RAR measure, we applied a “split-dose” exclusion in which a canceled order replaced by multiple orders with the same total dose was not flagged as an RAR event (eg, 40 mg retracted, two 20 mg reordered).

#### Varying measure parameters to minimize sample size needed for intervention trials

RAR measures provide valid, reliable, and robust outcome events to evaluate Health IT Safety interventions.^5^^,^^12^^,^^18^ Varying retract times and reorder times impact both the PPV and number of RAR events identified, with shorter intervals generally yielding a higher PPV that improves power, but fewer RAR events that decrease power. When developing new RAR measures, we seek to optimize each measure by identifying the retract and reorder times that would yield the best balance between PPV and number of events to provide the minimum sample size needed to power intervention trials using the RAR measure as the outcome. As illustrated in the example in Table 3, we can run sequential power analyses of a simulated trial that used RAR events as the primary outcome, varying combinations of retract times and reorder times to examine the effect of increasing PPV and decreasing number of RAR events on sample size. Smaller sample size requirements expedite transition from intervention testing to dissemination.

**Table 3.** Measure optimization.

		Based on validation data					Based on census data		
Retract time, minutes	Reorder time, minutes	True positives, *n*	Validated RAR events, *n*	PPV, %	95% LB	95% UB	Total RAR events, *n*	Total orders, *n*	Sample size, *n* (*D*_eff_ = 1)
5	2	72	80	0.90	0.81	0.96	5643	6 185 099	129 720
10	2	89	104	0.86	0.77	0.92	7818	6 185 099	105 198
15	2	109	126	0.87	0.79	0.92	9350	6 185 099	85 776
20	2	114	132	0.86	0.79	0.92	10 524	6 185 099	76 490
25	2	117	135	0.87	0.80	0.92	11 415	6 185 099	69 944
30	2	117	135	0.87	0.80	0.92	11 982	6 185 099	66 630
5	4	77	86	0.90	0.81	0.95	5994	6 185 099	123 600
10	4	94	110	0.85	0.77	0.91	8301	6 185 099	99 398
15	4	116	134	0.87	0.80	0.92	9940	6 185 099	80 550
20	4	122	141	0.87	0.80	0.92	11 197	6 185 099	71 578
25	4	125	144	0.87	0.80	0.92	12 136	6 185 099	65 540
30	4	125	144	0.87	0.80	0.92	12 746	6 185 099	62 398
5	6	77	87	0.89	0.80	0.94	6099	6 185 099	124 778
10	6	96	113	0.85	0.77	0.91	8446	6 185 099	99 016
15	6	119	138	0.86	0.79	0.92	10 115	6 185 099	79 868
20	6	125	145	0.86	0.80	0.91	11 413	6 185 099	70 820
25	6	128	148	0.86	0.80	0.92	12 374	6 185 099	64 826
30	6	128	148	0.86	0.80	0.92	12 998	6 185 099	61 710
5	8	78	89	0.88	0.79	0.94	6151	6 185 099	126 572
10	8	97	115	0.84	0.76	0.90	8516	6 185 099	99 836
15	8	120	140	0.86	0.79	0.91	10 197	6 185 099	80 332
20	8	126	147	0.86	0.79	0.91	11 507	6 185 099	71 176
25	8	129	150	0.86	0.79	0.91	12 478	6 185 099	65 128
30	8	129	150	0.86	0.79	0.91	13 112	6 185 099	61 974
5	10	78	89	0.88	0.79	0.94	6184	6 185 099	125 896
10	10	98	116	0.84	0.77	0.91	8560	6 185 099	98 958
15	10	121	141	0.86	0.79	0.91	10 252	6 185 099	79 684
20	10	127	148	0.86	0.79	0.91	11 568	6 185 099	70 616
25	10	130	151	0.86	0.80	0.91	12 549	6 185 099	64 598
**30**	**10**	**130**	**151**	**0.86**	**0.80**	**0.91**	**13 186**	**6 185 099**	**61 472**

An illustrative example of the effect of varying measure timing on PPV, RAR events, and sample size for the Wrong-Frequency RAR Measure, based on preliminary analysis. The optimal retract times and reorder times that result in the minimum sample size needed to power intervention trials using the RAR measure as the outcome is provided in the bolded last row.

Abbreviations: LB, lower bound; PPV, positive predictive value; RAR, retract-and-reorder; UB, upper bound.

For measures such as Wrong-Dose and Wrong-Frequency, we can also examine the effect of the magnitude of change between the initial order and reorder on PPV, as a greater magnitude may be more predictive of an error. For example, an insulin order change from 29 units to 30 units may be a false positive due to medication availability; however, a change from 3 units to 30 units is likely an error (*Measure Optimization* in Supplementary Material). Changes in measure specifications during measure optimization may prompt further measure validation with additional real-time clinician interviews.

### Measure utilization

Once RAR measures are validated and specifications are finalized, RAR order and interview data can be analyzed to understand the underlying mechanisms of errors (qualitatively) and the epidemiology of errors (quantitively) in healthcare settings. Together, these analyses improve our understanding and inform the design of targeted interventions to prevent these types of errors in at-risk patient populations. Furthermore, RAR events can also be used as an outcome measure to assess the effectiveness of implemented interventions. For example, the Wrong-Patient RAR measure was used to evaluate whether patient photographs displayed in the banner of an EHR is associated with a decreased rate of wrong patient ordering errors.^20^

#### Qualitative analysis: underlying reasons for errors

**Figure 4.**
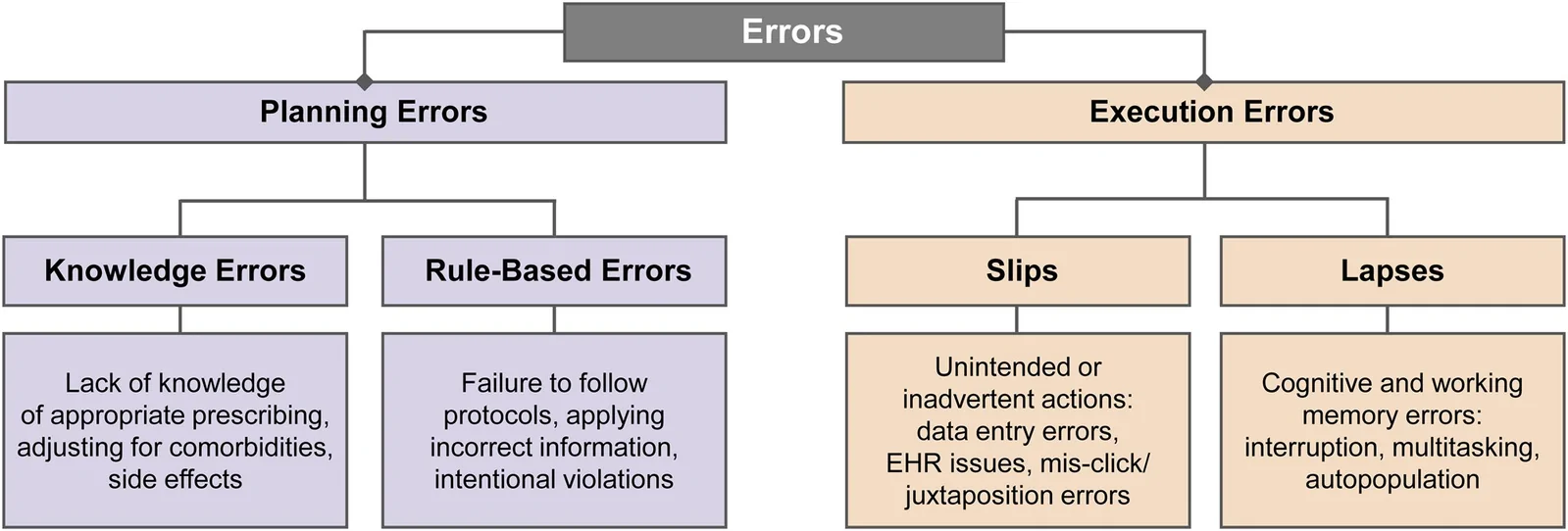
Coding scheme for qualitative analysis of reasons for errors. This framework distinguishes between planning errors, defined as errors in which the intended action is incorrect, and execution errors, defined as errors in which a correctly planned action is executed incorrectly. Planning errors are further categorized as knowledge errors (practical or clinical knowledge gaps), and rule-based errors (the incorrect application of or failure to apply a good rule or application of a bad rule). Execution errors consist of slips, defined as unintended actions (eg, clicking on the wrong medication), and lapses, defined as cognitive errors (eg, forgetting to hold medication based on abnormal lab values). Adapted from Ferner and Aronson.^40^

Using the validation data for each RAR measure, we conduct qualitative content analyses of clinician interview responses to describe the underlying reasons why errors occur.^42^ For medication safety RAR measures, we use a coding scheme derived from the Skills, Rules, Knowledge framework for classification of human error,^38^^,^^39^ adapted by Ferner and Aronson.^40^ (Figure 4). This framework distinguishes between planning errors, defined as errors in which the intended action is incorrect, and execution errors, defined as errors in which a correctly planned action is executed incorrectly. Planning errors are further categorized as knowledge errors (practical or clinical knowledge gaps) and rule-based errors (the incorrect application of or failure to apply a good rule or application of a bad rule). Execution errors consist of slips, defined as unintended actions (eg, clicking on the wrong medication), and lapses, defined as cognitive errors (eg, forgetting to hold medication based on abnormal laboratory values). This schema, originally developed for medication safety RAR measures, can also be used in the qualitative content analyses of other types of RAR measures.

When coding qualitative data, we use directed and emergent content analyses.^42^ Code definitions are standardized across error types and, as applicable, we develop emergent codes and subcodes specific to each error type. All event narratives are coded independently by 2 coders and disagreements are adjudicated by a third reviewer. This standardized approach enables us to compare the underlying mechanisms across error types to inform preventive strategies. Additionally, reasons for errors can be analyzed with respect to different retraction times and compared across error types.

#### Quantitative analysis: epidemiology of RAR events

The RAR measures can be used by healthcare organizations and researchers retrospectively over any specified period to describe the epidemiology of order errors, identify high-risk populations, and power outcome studies to rigorously evaluate interventions aimed at preventing order errors. For example, using the Wrong-Patient RAR measure, we demonstrated that the rate of wrong-patient errors in neonatal intensive care units (NICUs) was significantly greater than in general pediatric units and subsequently showed a significant reduction of these errors in the NICU after implementing a targeted intervention using the Wrong-Patient RAR measure as the outcome measure.^12–14^

## Discussion

We describe a framework for developing and validating measures of electronic order errors using tools readily accessible in most healthcare settings. Leveraging health IT to query the vast repository of existing clinical and electronic order data, we have presented an innovative and generalizable approach utilizing the RAR methodology to develop measures to study the epidemiology of electronic order errors, evaluate the root causes, and test system-level interventions aimed at preventing these errors. In addition, we highlight the iterative nature of our methods and underscore the lessons learned throughout this process. This roadmap, with illustrative examples, can be applied to develop and validate additional novel automated measures to detect a range of error types.

There is pressing need for feasible, comprehensive measurement of patient safety events. Despite the introduction of national regulations and pay-for-performance programs intended to improve patient safety, medical errors continue to occur at alarmingly high rates. Studies using 2018 data indicate that 25% of inpatients and 7% of outpatients experience adverse events, an estimated 23%-43% of which are preventable, and medication-related events are consistently the most frequent type of adverse event identified.^43–45^ Analysis of errors reported to the AHRQ’s Network of Patient Safety Databases estimated that over 2000 patients die each year from medication order errors.^34^ In response, the *Report to the President: A Transformational Effort on Patient Safety* advocates for patient safety as an urgent national public health issue and calls for the prevention of medical errors, recommending that “As many measures as possible should be generated from real-time automated electronic health data.”^46^

With the advent of RAR measures, research aimed at understanding and preventing order errors that was previously impracticable or impossible is now eminently feasible. In the early days of computerized provider order entry, wrong-patient orders were thought to result from juxtaposition errors (inadvertently clicking a name on a patient list adjacent to the intended patient) based on focus groups and surveys.^47–49^ However, our methodology used to validate the Wrong-Patient RAR measure enabled researchers to interview clinicians shortly after placing a wrong-patient order and elucidate the root cause of the error.^5^ These near-real time clinician interviews revealed that only 10.6% of wrong-patient order errors were juxtaposition errors, whereas interruption was the most common reason for these errors, accounting for 80.6%. The RAR measures mitigate recall bias by facilitating prompt discussions with ordering clinicians and introduce a new mechanism for exploring the underlying reasons for health IT errors in real time.

Testing large-scale, systemwide health IT interventions also was not feasible prior to the advent of RAR measures. For example, the Wrong-Patient RAR measure was used to power a randomized clinical trial of 12.1 million orders placed by 3356 clinicians to assess the risk of wrong-patient orders in an EHR configuration limiting clinicians to 1 record versus allowing up to 4 records open concurrently.^18^ Based on expert opinion, the ONC’s 2016 SAFER Guide recommended that the EHR limit the number of patient records that can be displayed on the same computer to one at a time.^10^ However, the clinical trial results did not demonstrate reduced rates of wrong-patient order errors among clinicians restricted to a single record.^18^ The ability of RAR measures to identify large volumes of outcome events allowed for a large randomized trial to generate evidence that did not support a national recommendation.

Future application of the RAR model could explore orders with more than one element changed when reordered (eg, dose and route), or orders placed then canceled by a different clinician (eg, an intern places the initial order, then a supervising resident cancels and places a corrected order). The RAR methodology can be applied to all types of electronic orders—medication, laboratory, radiology, and nursing orders—in the development and validation of many new RAR measures.

### Limitations

A limitation of RAR measures is that they do not capture errors that reach the patient and cause harm. However, near-miss errors are invaluable in patient safety research, as they occur up to 100 times more frequently than errors that reach the patient and thus provide a sufficient number of outcome events to power safety intervention studies.^50^ The use of near-miss errors to test safety improvements in healthcare is endorsed by every major organization dedicated to improving patient safety, including the Agency for Healthcare Research and Quality, Institute of Medicine, World Health Organization, Institute for Healthcare Improvement, and Joint Commission^50–53^ because near-miss errors follow the same causal pathway as errors that cause harm.^50^

## Conclusions

Utilizing illustrative examples, we have presented our unique process for measure conceptualization, development, implementation, validation, and optimization, and described how RAR measures can be utilized to examine the epidemiology of order errors, investigate the root causes in real-time, and rigorously evaluate the effectiveness of interventions. Overall, RAR measures facilitate unprecedented rapid-cycle evaluations of interventions to prevent order errors and drive evidence-based strategies. This report presents a framework intended to support health IT safety researchers to develop and validate additional RAR measures to detect order errors using electronic clinical and audit log data.

## Supplementary Material

ooag165_Supplementary_Data

## Data Availability

The data are available upon reasonable request from the authors.
