# Supplementary material for: A framework for developing, validating, and utilizing automated measures of order errors using the retract-and-reorder methodology
Source: JAMIA Open. 2026 Sep 9;9(5):ooag165. doi: 10.1093/jamiaopen/ooag165 (PMC13558070; doi:10.1093/jamiaopen/ooag165)
Supplement: Supplementary file 1 [file ooag165_supplementary_data.docx]

**Supplementary Materials**

Spector PR, Grauer A, Kneifati-Hayek JZ, Ranard BL, Applebaum JR, Fernandes Y, Salmasian H, Southern WN, Schechter CB, Hruby G, Cooke J, Kumaraiah D, Fertel B , Adelman JS. A framework for developing, validating, and utilizing automated measures of order errors using the retractand-reorder-methodology

**Retract-and-Reorder Glossary**

**False Positive:** are either non-errors or errors of a different type than the measure intended to capture. False positives that were non-errors resulted from order changes that were clinically equivalent (eg, due to medication availability, ease of administration, or based on the clinician’s judgment in which both the original order and the changed order were appropriate options), or order changes that were made because new clinical information became available after the initial order was placed (eg, lab results, change in clinical status). False positives that were errors of a different type are events in which the query captured errors but not of the type the measure was designed to capture (eg, the wrong-patient RAR measure captured a duplicate order that was canceled, and by happenstance followed by the same order being placed for another patient).

**Modified Order**: Some EHRs have a “modify function” that allow users to change an existing order. Functionally, modifying an order combines the actions of retracing an order and placing a new order, with some elements of the original order changed. Modified orders can be part of retract-and-reorder events.

**Ordering Clinician**: The ordering clinician is the person who signs the order in the EHR. Example 1: if a resident takes a verbal order from an attending physician, and enters and signs the order in the EHR, the resident is considered the ordering clinician. Example 2: if a medical student enters an order that is signed by a supervising resident, the supervising resident is considered the ordering clinician.

**Retract-and-Reorder (RAR) Event**: An RAR event occurs when an electronic order is placed for a patient (initial order), the order is then retracted (canceled) within XX minutes by the same ordering clinician (retracted order), and then the same order is placed by the same clinician within the next XX minutes (reorder) with an element of the order changed (patient, dose, etc.).

**RAR Measures**: automated measures that identify sequences of actions involving rapidly retracted orders that likely contain an error, and the orders placed immediately after the retraction, to identify health IT safety events that can be used to describe the epidemiology of order errors, investigate their root causes, and provide the outcome metric to test patient safety interventions.

**RAR Methodology or Model:** the broad concept that the sequence of events in which rapidly retracted orders represent errors, and the orders placed immediately after the corrected orders, can be applied to all types of electronic orders (eg. medication, lab, radiology, and nursing orders) to develop many RAR measures.

**RAR Rate**: the proportion calculated as the number of RAR events divided by the total number of orders of that type during a specified time period.

**Reordered Order:** An order that is placed within a brief, predefined time interval (eg, 10 minutes) immediately after a retracted order. In an RAR event, the reordered order is the corrected order.

**Retracted Order**: An order that is discontinued within a brief, predefined time interval (eg, 30 minutes). Retracted orders can be defined as orders that are “discontinued” or “cancelled.” In an RAR event, the retracted order is the erroneous order.

**True Positive (Error):** (measure-specified errors) were defined as order changes that captured the intended sequence of events specified by the measure (eg, wrong dose, wrong route, wrong frequency), and were made because placement of the initial order was unintentional, unplanned, due to lack of knowledge, due to failure to follow protocols, or was clinically inappropriate.

**Wrong-Patient RAR Event**: an order placed for a patient that is retracted (canceled) by the same clinician within 10 minutes, and then reordered by the same clinician for a different patient within the next 10 minutes.

**Wrong-Patient Order**: order placed for a patient (A) that was intended for a different patient (B).

**Wrong-Patient RAR Rate**: the proportion calculated as the number of Wrong-Patient RAR events divided by all orders during a specified time period.

**Wrong-Patient RAR Measure**: the Wrong-Patient RAR measure is an automated, validated, and reliable measure of wrong-patient orders. It was the first health IT safety measure endorsed by the National Quality Forum (NQF #2723). The measure identifies wrong-patient RAR events. (See definition of Wrong-Patient RAR Event)

Table S1. Variables for RAR Data Extraction

| **Variable** | **Coding/Description/Example** |
| --- | --- |
| **Clinician Variables** |  |
| User ID | Unique System Clinician ID |
| Clinician ID | Unique Clinician ID |
| Clinician Role | Attending, Fellow, Resident, PA, NP |
| Clinician Department | Pediatrics, Medicine, Surgery |
| **Patient Variables*** |  |
| Patient ID |  |
| Patient Age in years |  |
| Patient Age in days |  |
| Patient Gender | Male, Female, Not reported |
| Patient Race | Asian, Black/African American, Native American/Alaska Native, Native Hawaiian/Pacific Islander, White, More than One Race, not reported |
| Patient Ethnicity | Hispanic/Latino, Not Hispanic/Latino, Not reported |
| Patient Insurance Group | Medicare, Medicaid, Commercial, Self-Pay, Other |
| Visit Type | Inpatient, Outpatient, ED |
| Admission Date/Time | Date and time of admission |
| Discharge Date/Time | Date and time of discharge |
| Facility |  |
| Location/Unit |  |
| Service | Postpartum, NICU, Nursery |
| **Order Variables (Order, Reorder)** |  |
| Order ID | Unique order ID |
| Order Type | Medication, diagnostic, imaging, other |
| Order Entered Date/Time | Date/time of order |
| Order Retracted Date/Time | Date/time of retraction |
| Order Name | eg, ibuprofen oral |
| Order Form | eg, solution, injection |
| Order Dose | eg, 5 mg |
| Order Route | eg, oral |
| Order Frequency | eg, q12hr |
| Order Duration | eg, 7 days |
| Order PRN | Yes, No |
| Order Set | 0, 1 |
| Order Therapeutic Category | eg, analgesic |
| **RAR Variables** |  |
| Retract delay (time from initial order) | min |
| Reorder delay (time from retraction) | min |
| Error type (eg, wrong patient, wrong dose) |  |

*For the Wrong-Patient measure, these variables are collected for patient 1 and patient 2.

**Figure S1. RAR event details and interview guide embedded in measure-specific survey form.**

**Figure S2. Running RAR queries in real time for Measure Validation**

In the examples provided in the paper, RAR events were specified with a maximum retract time of 30 minutes and a maximum reorder time of 10 minutes. The RAR query looks back 70 minutes to ensure that no RAR events are missed, and to ensure that the reorder that completes the sequence of an RAR event is captured, even if the reorder occurs just before (Example 1) or just after (Example 2) new data is transferred to the RAR replica database. However, the majority of RAR events occur within short retract and reorder times (Example 3).

**Detailed Methods for Retract-and-Reorder (RAR) Measure Optimization**

RAR measures can be used to provide outcome events to evaluate the effectiveness of Health IT Safety interventions. When planning the evaluation of a Health IT Safety Intervention, both the positive predictive value (PPV) of an RAR measure and the number of events identified by the measure can impact the power of the research study. Varying retract times and reorder times can affect both the PPV and number of RAR events identified, with shorter retract and reorder times usually leading to higher PPVs but fewer RAR events. To optimize each measure, we used as the criterion *the minimum sample size needed to power intervention trials using the RAR measure as the outcome*. We thus examined the tradeoff between increasing PPV and decreasing prevalence of RAR events as determinants of sample size, by varying combinations of retract times and reorder times.

To calculate baseline prevalence of RAR events, we retrospectively ran the measures against all orders extracted from the data warehouse in a large healthcare system over a 1-year period. The numerator was the number of orders that qualified as RAR events for each measure, varying the cutoffs for retract times and reorder times. When applicable, the varying magnitude of change was also considered. For example, for the wrong-dose RAR measure, the difference between a change from 30 mgs to 3 mgs, vs a change from 4 mgs to 3 mgs, was considered. The denominator was the total number of orders for the error type (e.g., all orders, medication orders).

We then conducted a series of sample size calculations for a typical randomized controlled trial using RAR events as the outcome measure with the following assumptions: 1:1 allocation to intervention or control groups; a null hypothesis of no effect; an alternate hypothesis equivalent to a 50% reduction in the true error rate; using a two-tailed likelihood ratio test with α = 0.05 and power = 0.80. The actual sample size is the product of a design effect, which depends on the nesting structure and group allocation. The RAR measure specifications that require the smallest sample size for a simple logistic regression also require the smallest sample size for a hierarchical, multilevel analysis.

Assuming the intervention reduces true positives (errors) by 50%, the post-intervention number of RAR events would be N*0.5*PPV + N*(1-PPV) = N*(1 – 0.5*PPV), with an observed risk ratio of 1 – 0.5*PPV. We consider this risk ratio to be approximately equal to the corresponding odds ratio. For each measure, we used the baseline number of RAR events, and the calculated odds ratio to determine the sample size required to detect this effect. The timing that yielded the smallest sample size was selected as the optimal measure specification. We set the final specifications for all measures using this approach. An example of this analysis is provided in the paper, **Table 3**.
